# CCPLS reveals cell-type-specific spatial dependence of transcriptomes in single cells

**DOI:** 10.1093/bioinformatics/btad033

**Published:** 2023-02-16

*Bioinformatics*, Volume 38, Issue 21, 1 November 2022, Pages 4868–4877, https://doi.org/10.1093/bioinformatics/btac599

The authors apologize for the following errors. Notably, most of the errors are about representations in the paper and do not affect any results and conclusions.

(1) In Section 2.2.5 first paragraph, $w_{f,h,c}$ and $w_{f,h}$ should be $w_{f,h,c}^{\left(m\right)}$ and $w_{f,h}^{\left(m\right)}$, respectively.

(2) In Section 2.3.1 third paragraph of the PDF version, “$y_{i,h}^{'}\left(A\right)=\sum_{f}x_{i,f}^{\left(A\right)}w_{f,h}^{\left(A\right)}+\alpha e_{i,h}$” should be “${ý}_{i,h}^{\left(A\right)}=\sum_{f}x_{i,f}^{\left(A\right)}w_{f,h}^{\left(A\right)}+\alpha e_{i,h}$.”

(3) In Section 2.3.1 fourth paragraph, “Fig. S2a” should be “Fig. S2”.

(4) In Section 2.3.1 fifth paragraph, “Fig. S2” should be “Fig. S2a”.

(5) The title of Section 2.3.2 “Seqfish+ real dataset” should be “SeqFISH+ real dataset”.

(6) In Section 2.3.2 first paragraph, the hyperlink is corrected.

(7) In Section 2.3.3 first paragraph, we described Seq-Scope cell numbers as “4489 cells,” but the “4489 cells (subcellular spots)” is the accurate expression.

(8) In Section 3.1 second paragraph, “Based on the cluster assignment, we calculated ten indexes for performance evaluation (Fig. 2d).” should refer to “(Fig. 2d and e)” instead.

(9) In section 3.1 second paragraph, “All the calculated indexes for CCPLS results were greater than 0.97, which indicated that CCPLS estimated the prepared coefficients and the predefined HVG clusters with high performance.” should refer to “(Fig. 2d and e)”.

(10) In Section 3.1 second paragraph, “We also compared CCPLS with the existing method Giotto findICG, which was outperformed by CCPLS in this simulation experiment (Dries *et al.*, 2021b) (Fig. 2d).” should refer to “(Fig. 2e)” instead.

(11) In Section 3.1 third paragraph, “Condition 3” should be “Condition 9”.

(12) In Section 3.1 fourth paragraph, “VP” should be italic.

(13) In Section 3.2 first paragraph, “To demonstrate CCPLS with a real dataset, we applied CCPLS to the seqFISH+ real dataset (Eng *et al.*, 2019; Dries *et al.*, 2021b).” should refer to “(Fig. 3a)”.

(14) In Section 3.2 second paragraph, “We obtained the HVGs and the cell–cell communications for six cell types out of all 12 cell types (Fig. 3c and d and Supplementary Figs S2 and S3).” should refer to “(Fig. 3c and d and Supplementary Figs. S3 and S4)” instead.

(15) In Section 3.2 second paragraph, “We assigned the contributor cell types,” should refer to “(Supplementary Fig. S5)”.

(16) In Section 3.2 second paragraph, “which indicated that three cell types [astrocytes, oligodendro- cyte (Olig) and OPCs] cooperatively up-regulated HVG Clusters 1 and 2 in the OPCs.” should refer to “(Fig. 3d)”.

(17) In Section 3.2 third paragraph, “Fig. S3” should be “Fig. S6”.

(18) In Section 3.2 third paragraph, “The top 10 GO terms are indicated in Figure 4e (Fig. 3e).” should refer to “Figure 3e” only.

(19) In Section 3.2 fourth paragraph, “Fig. S4” and “Fig. S5” should be “Fig. S7” and “Fig. S8”, respectively.

(20) In Section 3.2 fifth paragraph, “SeqFISH+” should be “seqFISH+”.

(21) In Section 3.2 fifth paragraph, “Fig. S6” and “Fig. S7” should be “Fig. S9” and “Fig. S10”, respectively.

(22) In Section 3.2 sixth paragraph, “Fig. S3 and S8-S10” should be “Figs. S3-S6”.

(23) In Section 3.2 sixth paragraph, the hyperlink is corrected.

(24) In Section 3.3 first paragraph, “For the further demonstration of CCPLS using a real dataset, we applied CCPLS to the Seq-Scope real dataset (Cho *et al.*, 2021).” should refer to “(Fig. 4a)”.

(25) In Section 3.3 second paragraph, “Figs S6 and S7” should be “Figs. S11 and S12”.

(26) In Section 3.3 second paragraph, “We assigned the contributor cell types,” should refer to “(Supplementary Fig. S13)”.

(27) In Section 3.3 second paragraph, “which indicated that the immature B cell and the fibroblast cell types cooperatively down-regulated HVG Cluster 1 in the immature B cell.” should refer to “(Fig. 4d)”.

(28) In Section 3.3 fourth paragraph, “Fig. S11” should be “Fig. S14”.

(29) In Section 3.3 fifth paragraph, “Fig. S12” and “Fig. S13” should be “Fig. S15” and “Fig. S16”, respectively.

(30) In Section 3.3 sixth paragraph, “Figs S14-S17” should be “Figs. S11-13 and S17”.

(31) In Section 3.3 sixth paragraph, the hyperlink and typo were corrected.

(32) Text S1, the variables and functions were changed to be italic.

(33) In Text S1 first paragraph, we described as follows:

 wf,h,c'm=wf,h,cm       if qf,cm<α ∨qh,cm<αwf,h,c'm =0           otherwise,

but the following is correct:

 wf,h,c'm=wf,h,cm       if qf,cm<α ∧qh,cm<αwf,h,c'm =0           otherwise.

(34) In Text S1 second paragraph, “CCPLS uses the coefficients ${w_{f,h}}^{\left(m\right)}$ which are statistically non-significant genes in all the neighboring cell types f in the step (i) as a null distribution.” should be “For each neighboring cell type f, CCPLS uses the coefficients ${w_{f,h}}^{\left(m\right)}$ which are statistically non-significant genes in the step (i) as a null distribution.”

(35) In Text S2 Section 2.1 fourth paragraph, we defined the VP as follows:

$$\mathrm{VP}=\frac{1}{H}\Sigma_{h}\{\frac{\mathrm{var}\left(x_{i,f}w_{f,h}^{\left(A\right)}\right)}{\mathrm{var}\left(x_{i,f}w_{f,h}^{\left(A\right)}+\alpha e_{i,h}\right)}\},$$

but the following is correct:

$$VP=\frac{1}{H}\Sigma_{h}\left\{\frac{\mathrm{var}\left(y_{h}^{\mathrm{sim}}\right)}{\mathrm{var}\left(y_{h}^{\mathrm{tot}}\right)}\right\},$$

where $y_{h}^{\mathrm{sim}}=(\sum_{f}x_{1,f}^{\left(A\right)}w_{f,h}^{\left(A\right)},\ldots ,\sum_{f}x_{N,f}^{\left(A\right)}w_{f,h}^{\left(A\right)})$ and $y_{h}^{\mathrm{tot}}=(y_{1,h}^{'(A)},\ldots ,y_{N,h}^{'(A)})$.

(36) In Figs. S3-S5, we added results of L6 eNeuron, which should be displayed originally.

(37) In Fig. S13, results of B cell-IgG were updated.

## Supplementary Material

btad033_Supplementary_Data

