# Supplementary material for: CCPLS reveals cell-type-specific spatial dependence of transcriptomes in single cells
Source: Bioinformatics. 2022 Sep 5;38(21):4868–77. doi: 10.1093/bioinformatics/btac599 (PMC9620831; doi:10.1093/bioinformatics/btac599)
Supplement: btac599_Supplementary_Data [file btac599_supplementary_data.pdf]

Supplementary Information

CCPLS reveals cell-type-specific spatial dependence of transcriptomes in single cells

Takaho Tsuchiya, Hiroki Hori and Haruka Ozaki*

∗To whom correspondence should be addressed.

Contents:

Text S1. Filtering of coefficients in CCPLS.

Text S2. Additional descriptions of datasets.

Table S1. Computational methods for spatial transcriptome data.

Figure S1. Evaluation using the simulated datasets across parameters.

Figure S2. Evaluation using the noise derived from gamma distribution.

Figure S3. Heat map of all the cell types in the seqFISH+ real dataset.

Figure S4. Bipartite graph of all the cell types in the seqFISH+ real dataset.

Figure S5. Classification of the contributor cell types in the seqFISH+ real dataset.

Figure S6. Gene Ontology (GO) enrichment of all the cell types in the seqFISH+ real dataset..

Figure S7. Spatial distribution of cluster 1 (*Tmem98*) and 2 (*Mag*, *Abl1*, *Dock6*, and *Kndc1*) genes expression in Oligodendrocyte Precursor cells (OPCs).

Figure S8. Relationship between the gene expression and the nearest distance to neighboring cell types.

Figure S9. Comparison between Giotto findICG and CCPLS in the seqFISH+ real dataset.

Figure S10. Number of overlaps between genes of GO 'glial cell differentiation,' genes detected by CCPLS, and genes detected by Giotto findICG.

Figure S11. Heat map of all the cell types in the Seq-Scope real dataset.

Figure S12. Bipartite graph of all the cell types in the Seq-Scope real dataset.

Figure S13. Classification of the contributor cell types in the Seq-Scope real dataset.

Figure S14. Spatial distribution of *Gpx1* expression in B cell-Immature.

Figure S15. Comparison between Giotto findICG and CCPLS in the Seq-Scope real dataset.

Figure S16. Number of overlaps between genes of GO 'epithelial cell development,' genes detected by CCPLS, and genes detected by Giotto findICG.

Figure S17. Gene Ontology (GO) enrichment of all the cell types in the Seq-Scope real dataset.

Text S1. Filtering of coefficients in CCPLS

In filtering step (i), CCPLS calculates *p*-values for $w_{f,h,c}^{(m)}$ of each component c, which is an element of $w_{f,h}^{(m)}$ and is obtained by PLS regression modeling. The *p*-values are calculated by *t*-tests of factor loadings (Yamamoto *et al*., 2014), which correspond to the *p*-values of the Pearson correlation coefficient calculated by

$$corr\left(\boldsymbol{x}_f^{(m)}, \boldsymbol{t}_c^{(m)}\right) = \frac{\boldsymbol{x}_f^{(m)} \mathbf{t}_c^{(m)} / (N^{(m)} - 1)}{\sqrt{var\left(\boldsymbol{x}_f^{(m)}\right)} \sqrt{var\left(\boldsymbol{t}_c^{(m)}\right)}} \ ,$$

$$corr\left(\boldsymbol{y}_h^{(m)}, \boldsymbol{u}_c^{(m)}\right) = \frac{\boldsymbol{y}_h^{(m)} \boldsymbol{u}_c^{(m)} / (N^{(m)} - 1)}{\sqrt{var\left(\boldsymbol{y}_h^{(m)}\right)} \sqrt{var\left(\boldsymbol{u}_c^{(m)}\right)}} \ ,$$

where $corr()$ and $var()$ denote calculations of a Pearson correlation coefficient and a variance, respectively. The vectors $\boldsymbol{x}_f^{(m)}$ and $\boldsymbol{y}_h^{(m)}$ are preprocessed scores of neighboring cell type f and preprocessed expression values of HVG h of cells i within cell type m. The vectors $\boldsymbol{t}_c^{(m)}$ and $\boldsymbol{u}_c^{(m)}$ are scores of c-th component obtained by PLS regression modeling. These *p*-values are false discovery rate (FDR)-adjusted as $q_{f,c}^{(m)}$ and $q_{h,c}^{(m)}$ by the Benjamini–Hochberg (BH) method, respectively (Benjamini and Hochberg, 1995). CCPLS filters out the coefficient $w_{f,h,c}^{(m)}$ whose adjusted *p*-values $q_{f,c}^{(m)}$ or $q_{h,c}^{(m)}$ are greater than or equal to α and then returns the coefficient $w'^{(m)}_{f,h} = \Sigma_{\mathrm{c}} w'^{(m)}_{f,h,c}$ as follows:

$$\begin{cases} w'^{(m)}_{f,h,c} = w^{(m)}_{f,h,c} & \text{if } q^{(m)}_{f,c} < \alpha \wedge q^{(m)}_{h,c} < \alpha \\ w'^{(m)}_{f,h,c} = 0 & \text{otherwise} \end{cases} .$$

In this study, we set α to 0.05.

In filtering step (ii), CCPLS filters out the statistically non-significant coefficient $w'^{(m)}_{f}$ by using a non-parametric test. For each neighboring cell type f, CCPLS uses the coefficients $w_{f,h}^{(m)}$ which are statistically non-significant genes in the step (i) as a null distribution. For each neighboring cell type f, CCPLS calculates the adjusted *p*-values $q'^{(m)}_{f,h}$ by the BH method (Benjamini and Hochberg, 1995). For each neighboring cell type f, CCPLS filters out the coefficient $\mathrm{w}'^{(m)}_{f,h}$ whose adjusted *p*-values $q'^{(m)}_{f,h}$ are greater than or equal to α and then returns $w''^{(m)}_{f,h}$ as follows:

$$\begin{cases} w''^{(m)}_{f,\mathrm{h}} = w'^{(m)}_{f,\mathrm{h}} & \text{if } q'^{(m)}_{f,h} < \alpha \\ w''^{(m)}_{f,\mathrm{h}} = 0 & \text{otherwise} \end{cases} .$$

Text S2. Additional descriptions of datasets

S2.1 Simulated dataset

We prepared the cell type label vector $\boldsymbol{L}$ by substituting cell types A-D into the cell type label vector of the seqFISH+ real dataset as follows:

- A: L5 eNeuron
- B: L6 eNeuron
- C: Olig
- D: The other nine cell types

In Section 3.1, we assigned the correspondence between the estimated and predefined highly variable gene (HVG) clusters based on whether greater than half of genes had estimated coefficients corresponding to each flag in the following table, respectively. We assigned clusters to the group "others" if they were not assigned to any of the predefined clusters.

Neighboring cell type	Flag of predefined cluster 1	Flag of predefined cluster 2	Flag of predefined cluster 3	Flag of predefined cluster 4
A	Significant	Not significant	Not significant	Not significant
B	Not significant	Significant	Not significant	Not significant
C	Significant	Not significant	Significant	Not significant
D	Not significant	Not significant	Not significant	Not significant

For Giotto findICG, we assigned HVG cluster 1 if the sender cell type was "A" or "C," and HVG clusters 2-4 if the sender cell type was "B," "C," or none-detected, respectively.

For CCPLS, based on these assignments, we calculated the adjusted Rand index. For CCPLS, we calculated Pearson correlation coefficients from estimated coefficients not divided by each assignment. For CCPLS and Giotto findICG, based on these assignments, we calculated precision and recall for each HVG cluster. We also calculated the index of variance proportion (VP) as follows:

$$VP = \frac{1}{H}\Sigma_h \left\{\frac{var\left(\boldsymbol{y}_h^{\mathrm{sim}}\right)}{var\left(\boldsymbol{y}_h^{\mathrm{tot}}\right)}\right\},$$

where $\boldsymbol{y}_h^{\mathrm{sim}} = \left(\Sigma_f x_{1,f}^{(A)} w_{f,h}^{(A)}, \dots, \Sigma_f x_{N,f}^{(A)} w_{f,h}^{(A)}\right)$ and $\boldsymbol{y}_h^{\mathrm{tot}} = \left(y_{1,h}'^{(A)}, \dots, y_{N,h}'^{(A)}\right)$.

S2.2 SeqFISH+ real dataset

In Section 3.2, we assigned the contributor cell types, which were the common neighboring cell types for each HVG cluster. If the coefficient vector $\boldsymbol{w}_f^{(m)} = (w_{f,1}^{(m)}, \dots, w_{f,H}^{(m)})$ was significant in more than half of the genes relative to the neighboring cell type f, we assigned it as the contributor cell type.

We performed Gene Ontology (GO) enrichment analysis of biological processes for each HVG cluster. If the distinct HVG clusters within the same cell type had the same contributor cell types, the genes were merged. We performed a hypergeometric test and extracted the significant GO terms with adjusted p- values less than 0.05 based on the BH method (Benjamini and Hochberg, 1995). We selected background genes whose raw expression values were greater than 0 within each cell type.

Table S1. Computational methods for spatial transcriptome data

Method	Reference	Effect of neighboring cell types	MIMO system	Descriptions
CCPLS	This study	Consider	Consider	Estimation of regulation on highly variable genes by multiple neighboring cell types based on PLS regression modeling.
Giotto findICG	Dires et al., 2021b	Consider	Not consider	Estimation of genes influenced by neighboring cell type based on spatial permutation test.
Giotto spatCellCellcom	Dires et al., 2021b	Consider	Not consider	Estimation of genes influenced by neighboring cell type based on permutation test.
SptialDE	Svensson et al., 2018	Not consider	Not consider	Estimation of spatially variable genes based on Gaussian process regression modeling of spatial gene expression.
TrendSceek	Edsgärd et al., 2018	Not consider	Not consider	Estimation of spatially variable genes based on marked point process modeling of spatial gene expression.
SPARK-X	Zhu et al., 2021	Not consider	Not consider	Estimation of spatially variable genes based on spatial kernels and non-parametric modeling of spatial gene expression.
Giotto BinSpect	Dries et al., 2021b	Not consider	Not consider	Estimation of spatially variable genes based on enrichment analysis of spatially high expression cells after binarization.
Giotto SilhouetteRank	Dries et al., 2021b	Not consider	Not consider	Estimation of spatially variable genes by silhouette score per gene based on spatial distribution of two cells.
SVCA	Arnol et al., 2019	Not consider	Not consider	Estimation of spatial variance sources of individual gene based on Gaussian process regression modeling of spatial gene expression.
SpaGCN	Hu et al., 2021a	Not consider	Not consider	Estimation of spatial domain and genes expression patterns based on graph convolutional network analysis.
MEFISTO	Velten et al., 2022	Not consider	Not consider	Estimation of spatial gene expression patterns based on factor analysis.
MISTy	Tanevski et al., 2022	Not consider	Not consider	Estimation of gene-gene relationships from different spatial views: intrinsic, local niche view, the broader, tissue view, or others.

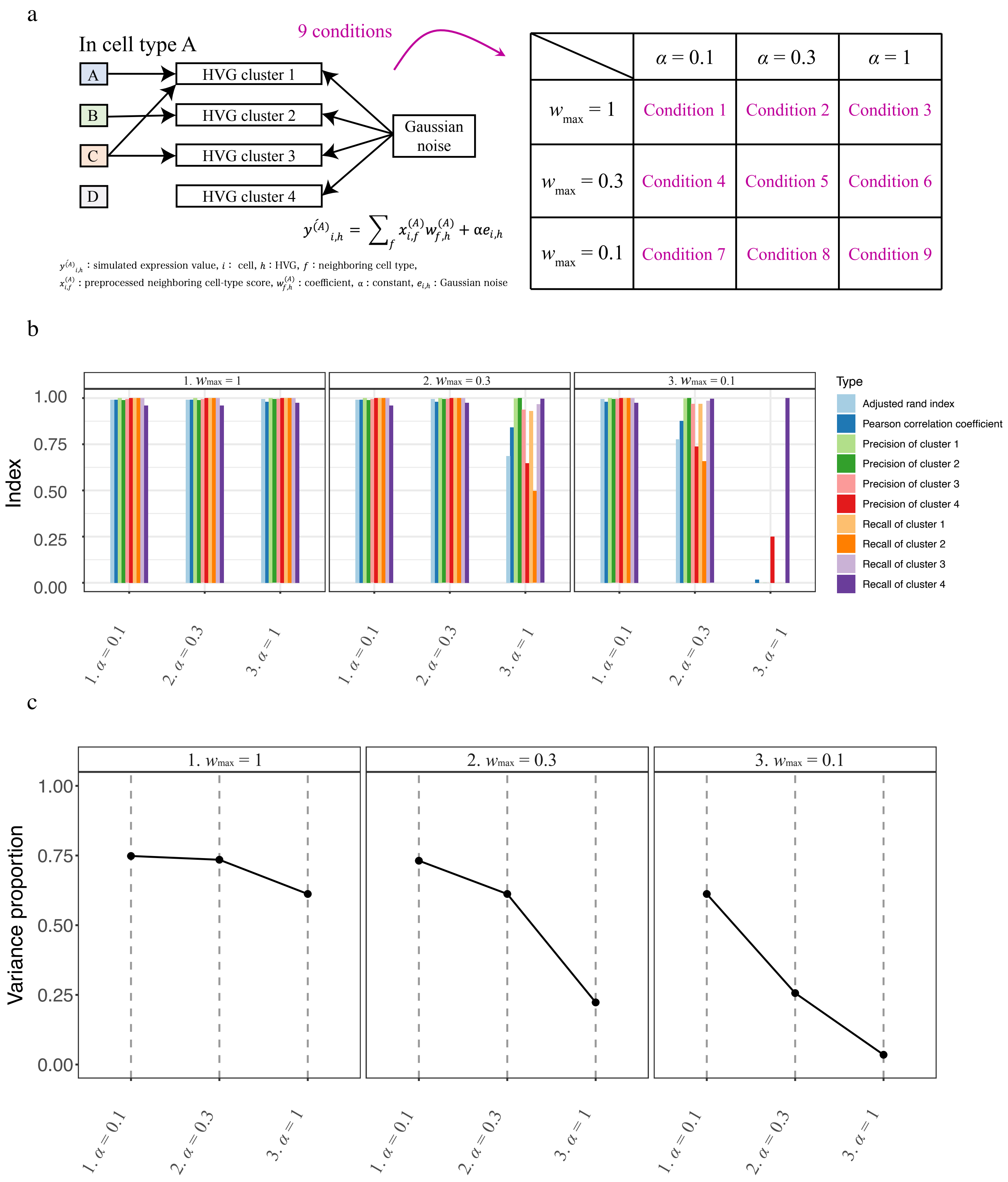

Figure S1. Evaluation using the simulated datasets across parameters. (a) Schematic illustration of the simulation settings with the changed parameters. Note that condition 3 corresponds to the condition in Figure 2. (b) Performance indexes in each condition. The value of each index is indicated along the y-axis, while each condition is arranged along the x-axis grouped within each w_{max} value. The color indicates the index type. (c) Variance proportion in each condition. The variance proportion is indicated along the y-axis, while each condition is arranged along the x-axis grouped within each w_{max} value.

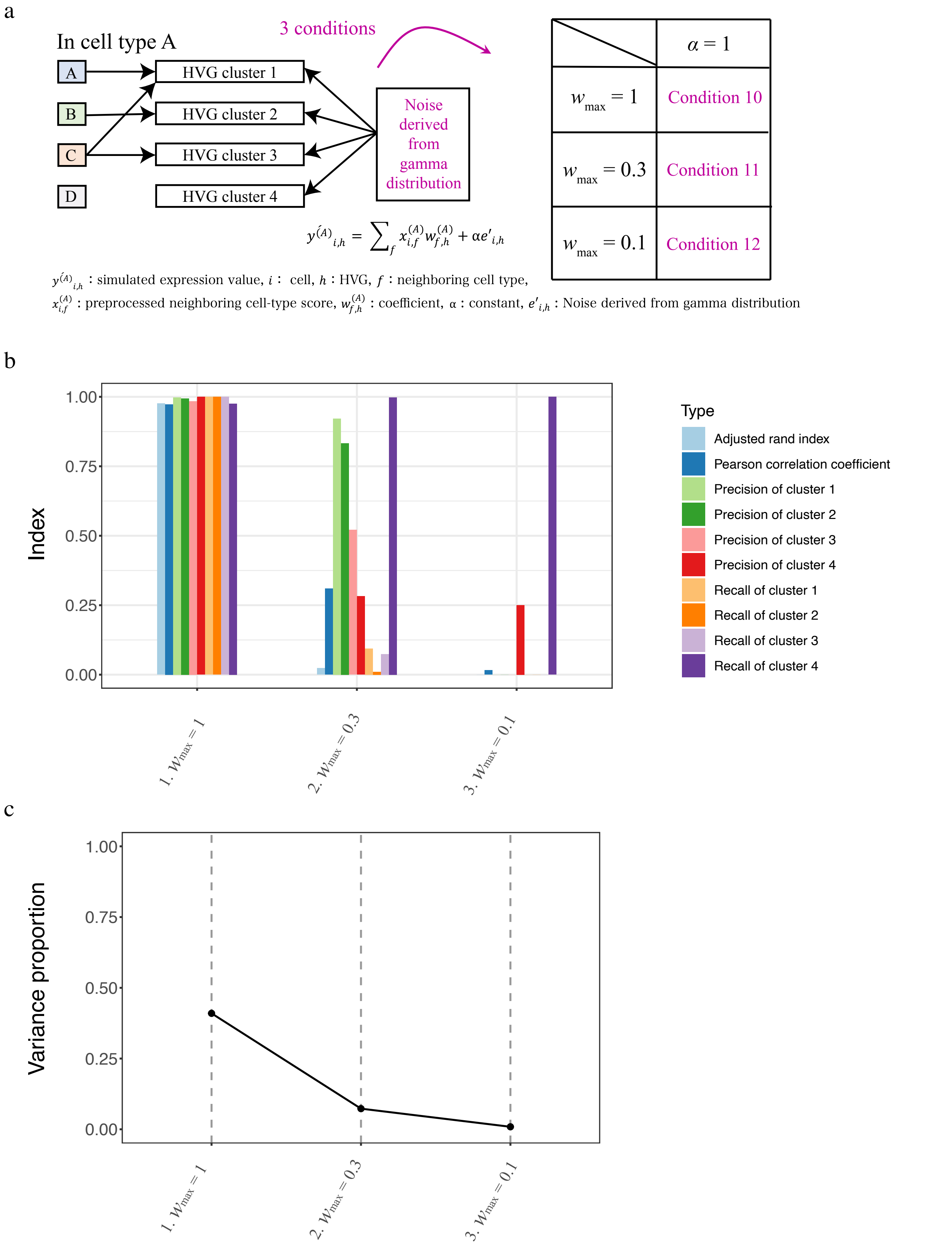

Figure S2. Evaluation using the noise derived from gamma distribution. (a) Schematic illustration of the simulation settings with the changed parameters. Note that we only replaced the Gaussian noise with the noise derived from gamma distribution compared with the Figure 2 and Figure S1. (b) Performance indexes in each condition. The value of each index is indicated along the y-axis, while each condition is arranged along the x-axis. The color indicates the index type. (c) Variance proportion in each condition. The variance proportion is indicated along the y-axis, while each condition is arranged along the x-axis grouped within each w_{max} value.

Apllication to the seqFISH+ real dataset:

Color of heatmap: coefficient

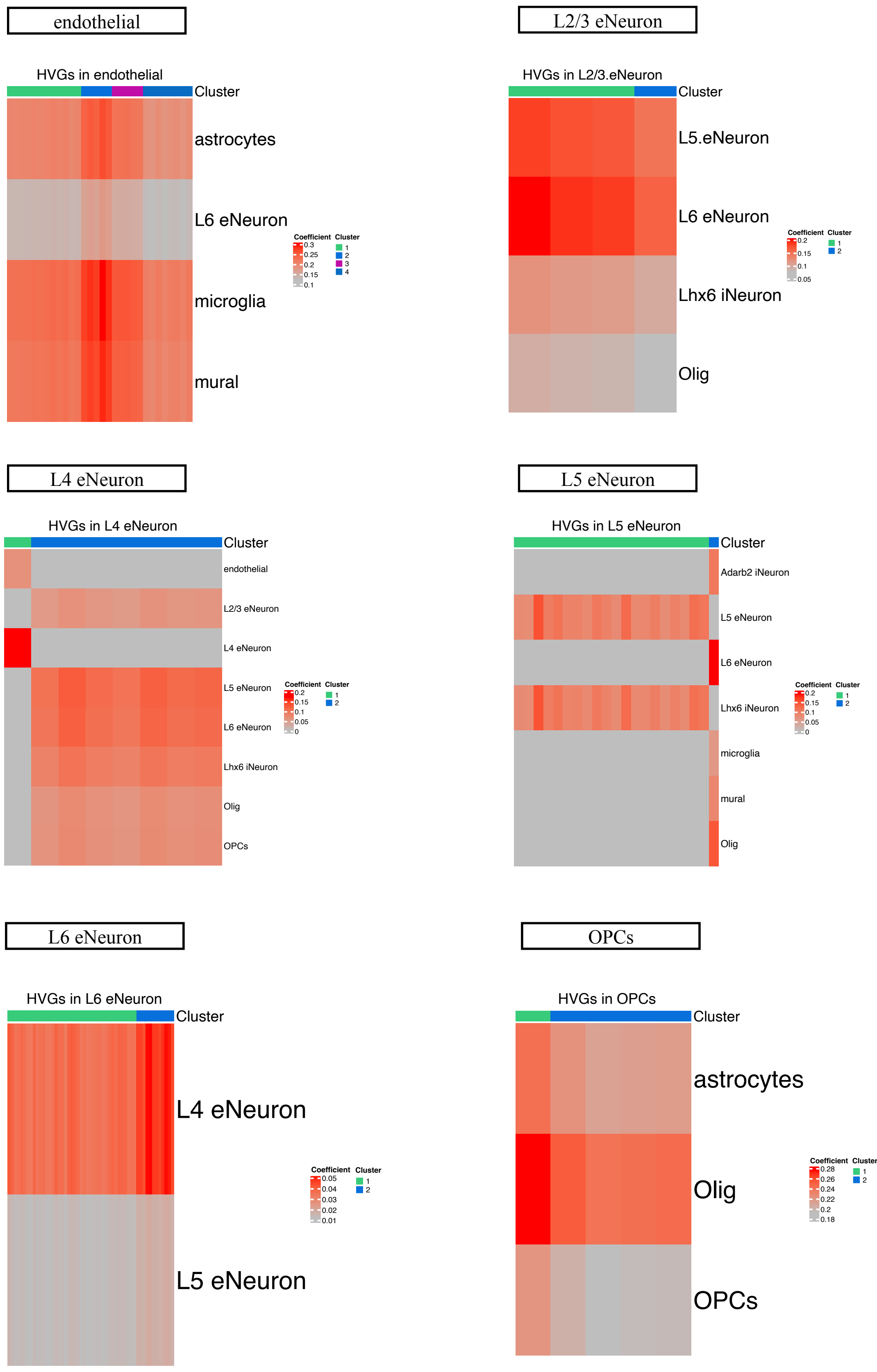

Figure S3. Heat map of all the cell types in the seqFISH+ real dataset. Rows and columns correspond to neighboring cell types and highly variable genes (HVGs), respectively. The color of the heat map indicates the coefficient. The heatmap of oligodendrocyte precursor cells (OPCs) is the same as that shown in Figure 3c.

Application to the seqFISH+ real dataset:

Width of edge: averaged coefficients

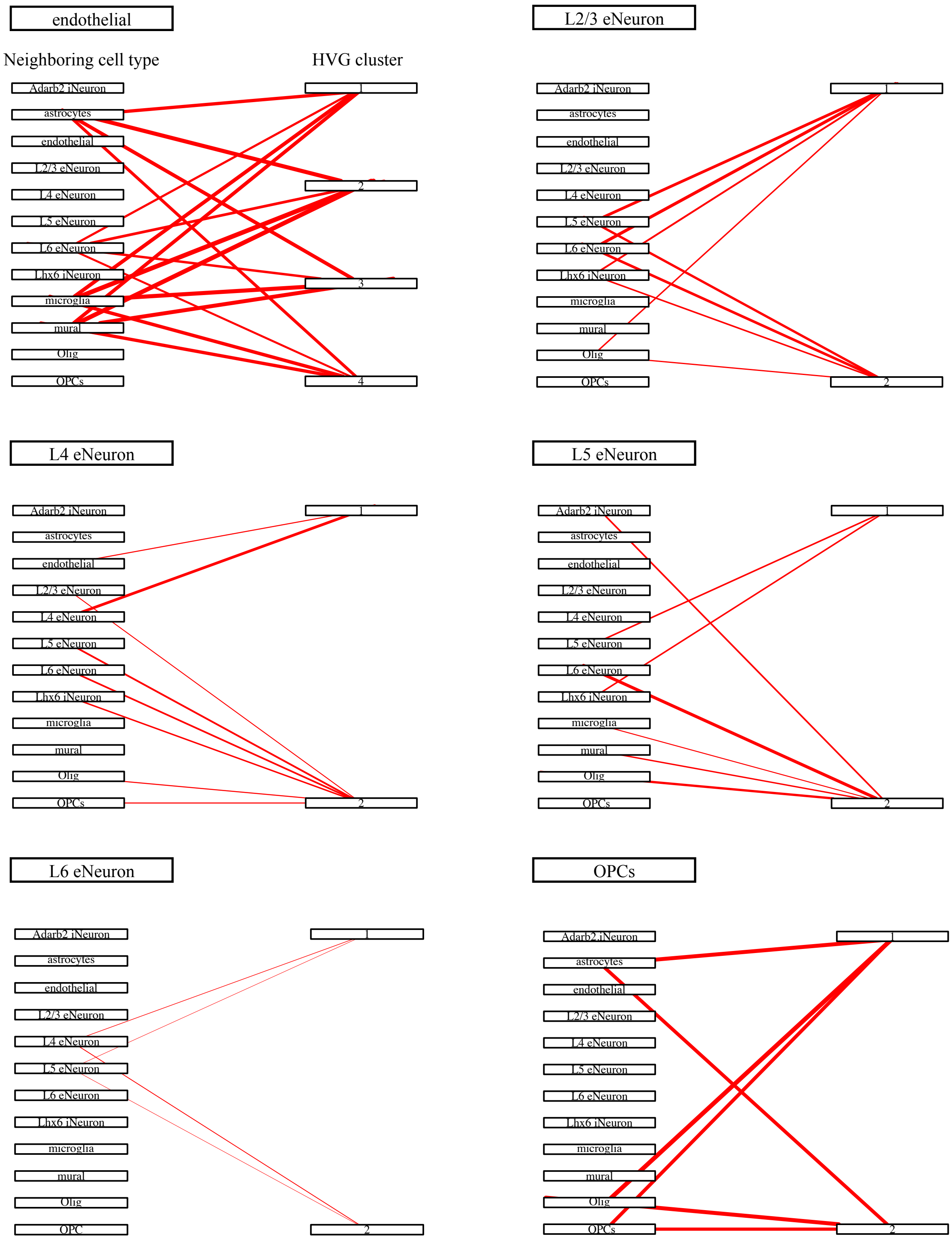

Figure S4. Bipartite graph of all the cell types in the seqFISH+ real dataset. The width of each edge indicates the averaged coefficients in each combination of highly variable gene (HVG) clusters and neighboring cell types. The bipartite graph of oligodendrocyte precursor cells (OPCs) is as the same as that shown in Figure 3d.

Application to the seqFISH+ real dataset:

Red: contributor cell type   Blue: non-contibutor cell type Row: neighboring cell type   Column: HVG cluster

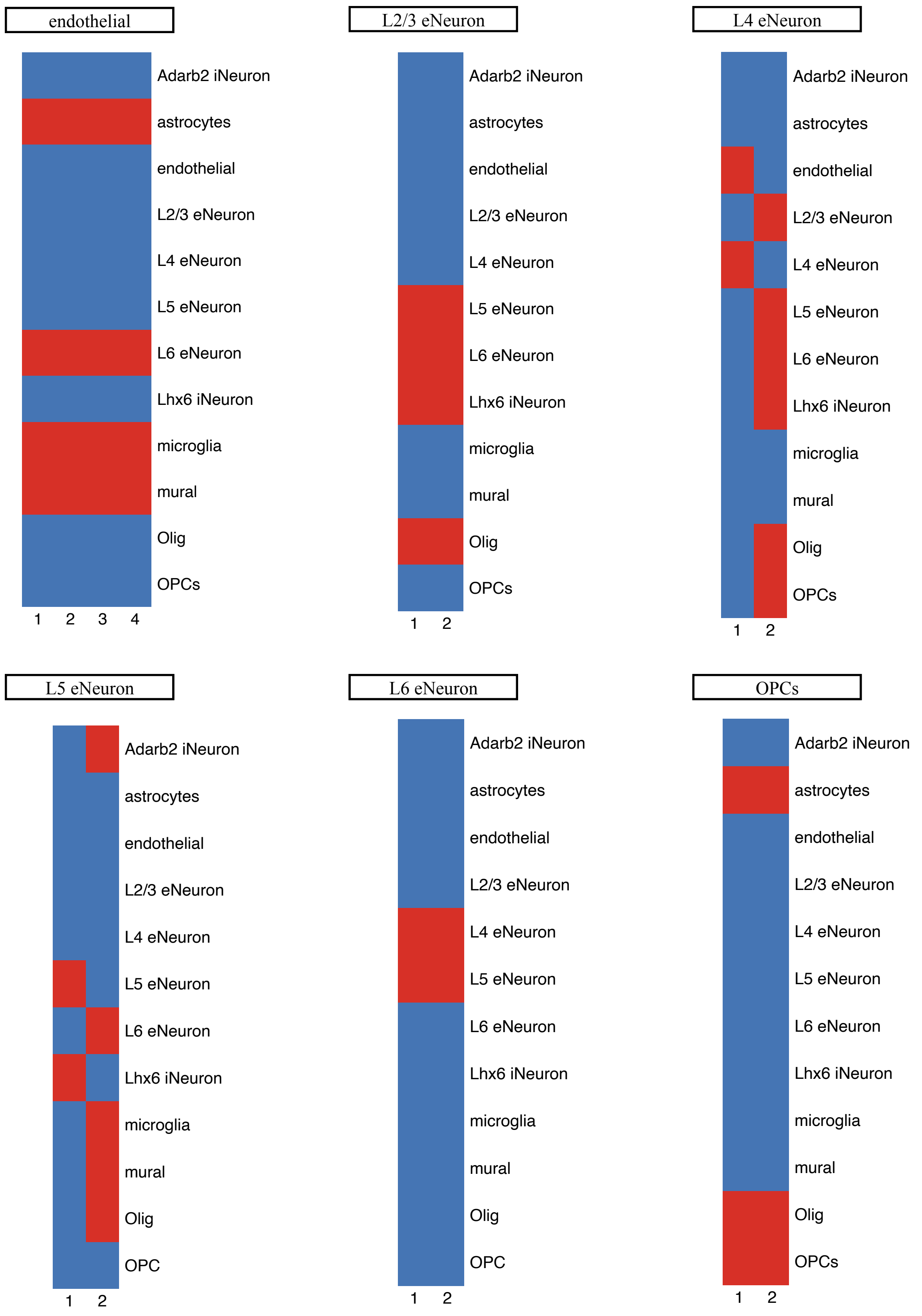

Figure S5. Classification of the contributor cell types in the seqFISH+ real dataset. The color of the heat map corresponds to the binary value indicating whether the neighboring cell type is a contributor cell type or not. Red and blue indicate the contributor and non-contributor cell types, respectively. Rows and columns correspond to cell types and highly variable gene (HVG) clusters, respectively.

<u>Application to the seqFISH+ real dataset:</u>

Row: GO term Column: gene count
Color of bar graph: adjusted *p*-value

L2/3 eNeuron

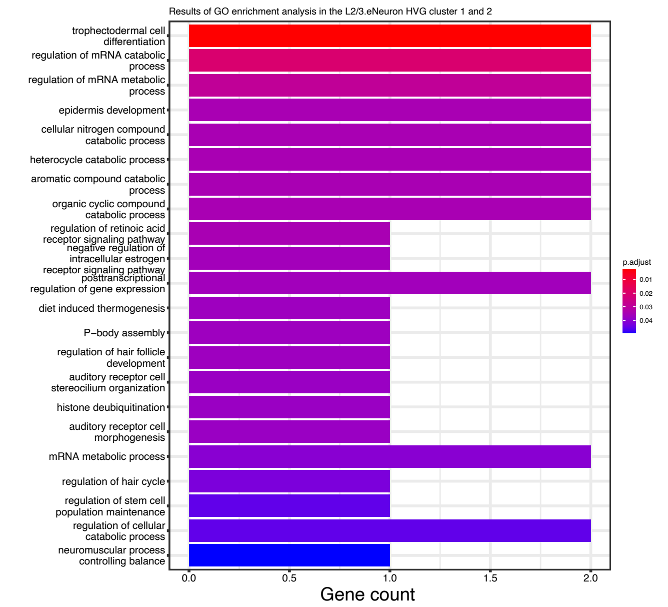

L4 eNeuron

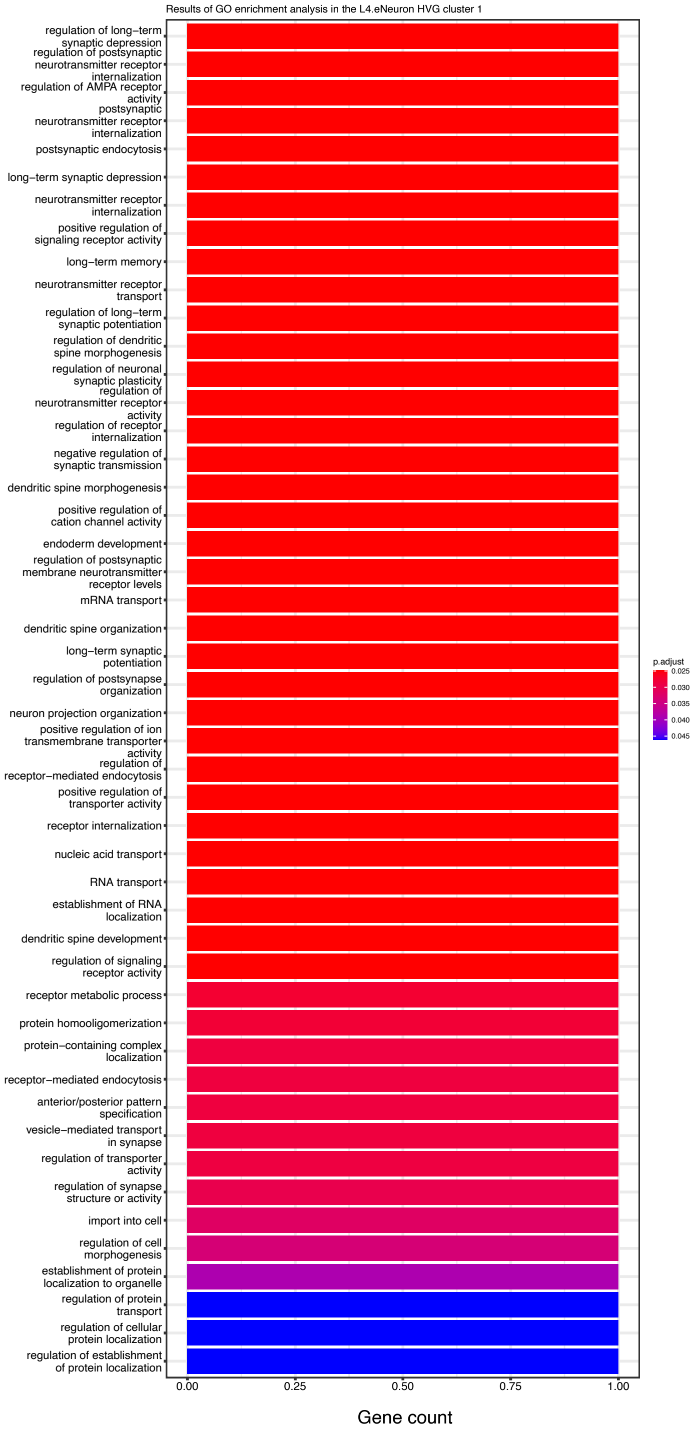

L5 eNeuron

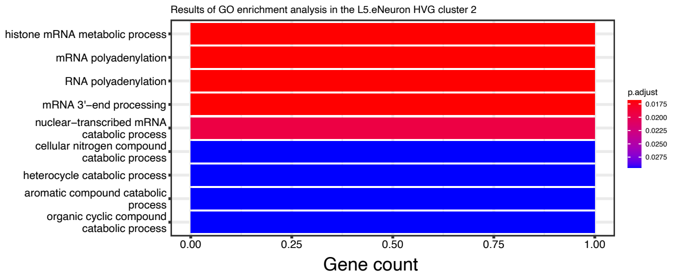

OPCs

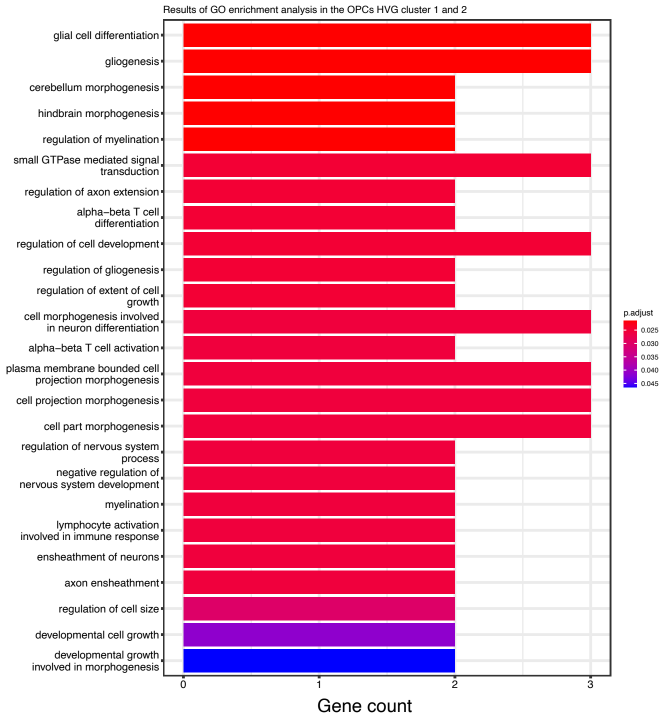

Figure S6. Gene Ontology (GO) enrichment of all the cell types in the seqFISH+ real dataset.

Apllication to the seqFISH+ real dataset:

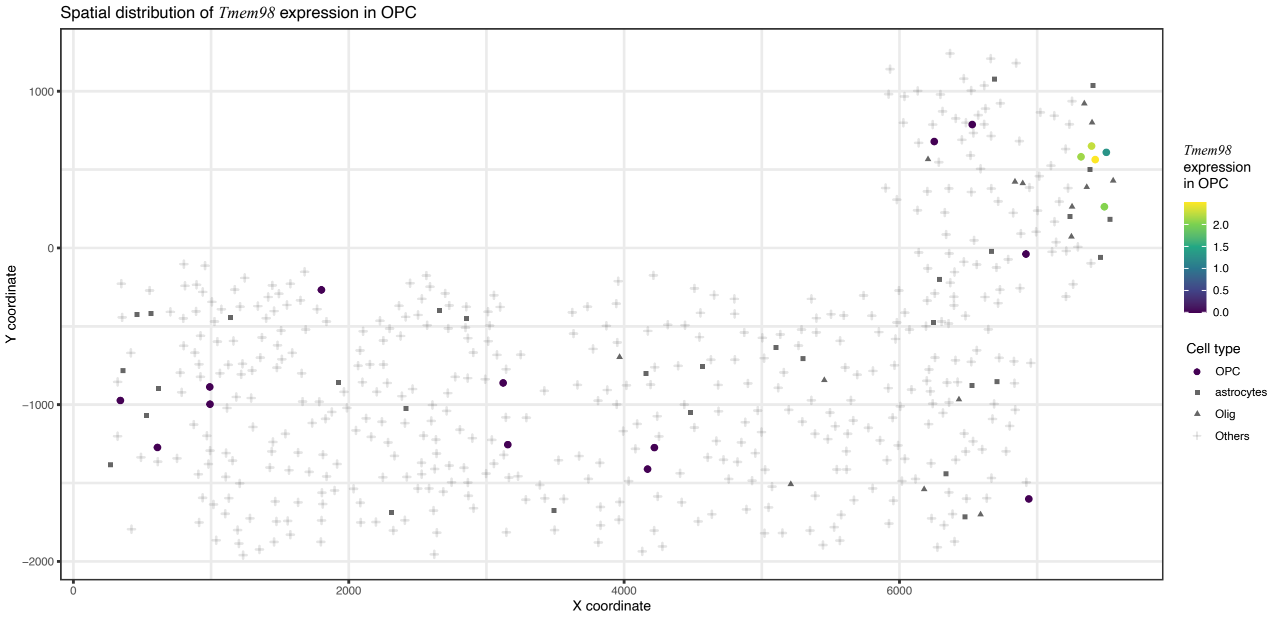

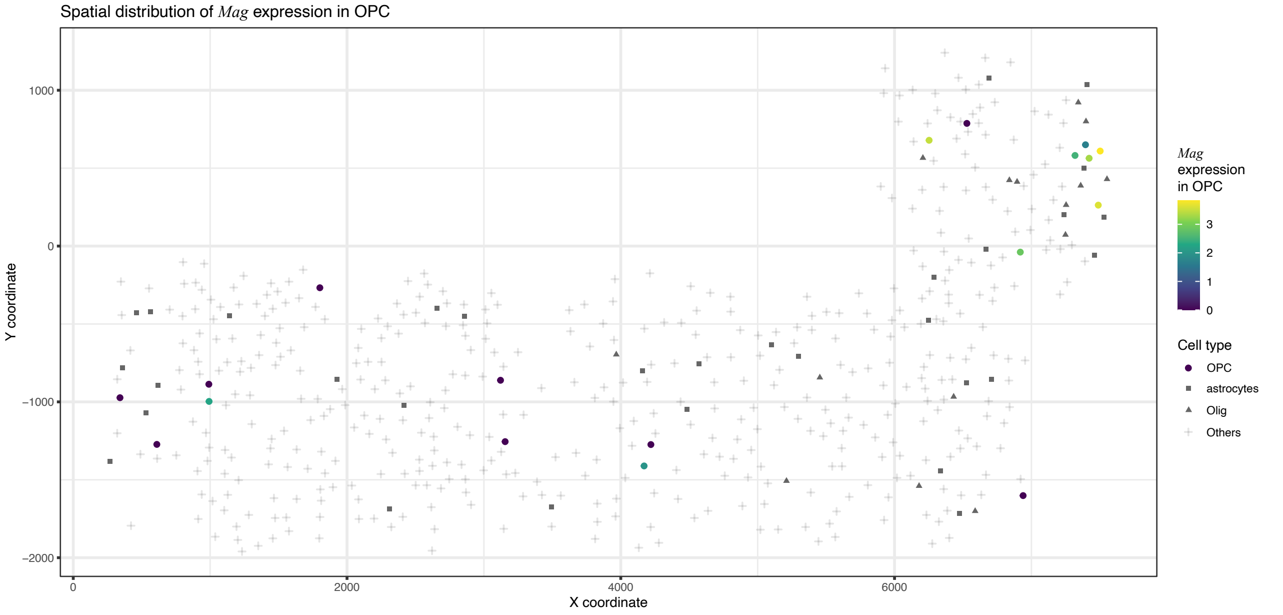

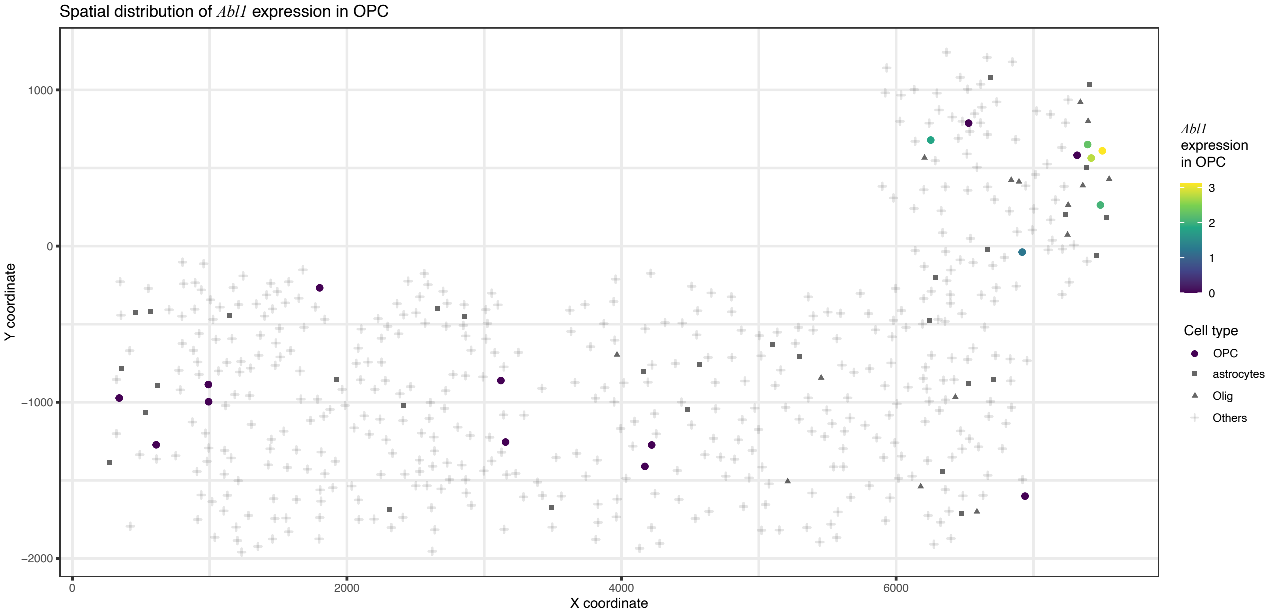

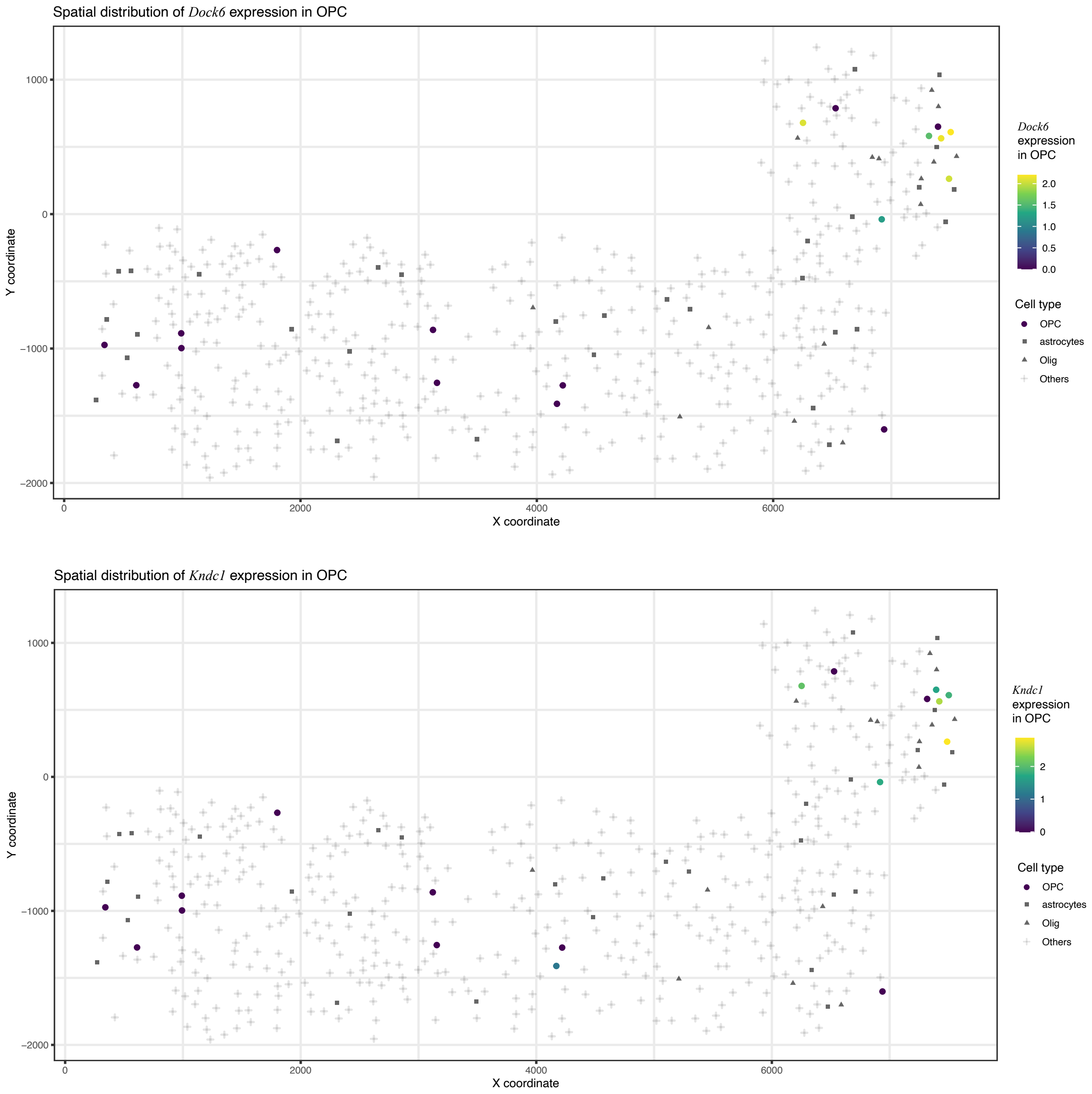

Figure S7. Spatial distribution of the cluster 1 (*Tmem98*) and 2 (*Mag*, *Abl1*, *Dock6*, and *Kndc1*) genes expression in Oligodendrocyte Precursor cells (OPCs). Each panel indicates spatial distribution of each gene. The shapes indicate cell types. The color in the circles indicates values of gene expression.

Apllication to the seqFISH+ real dataset:

Tmem98 | *Mag*

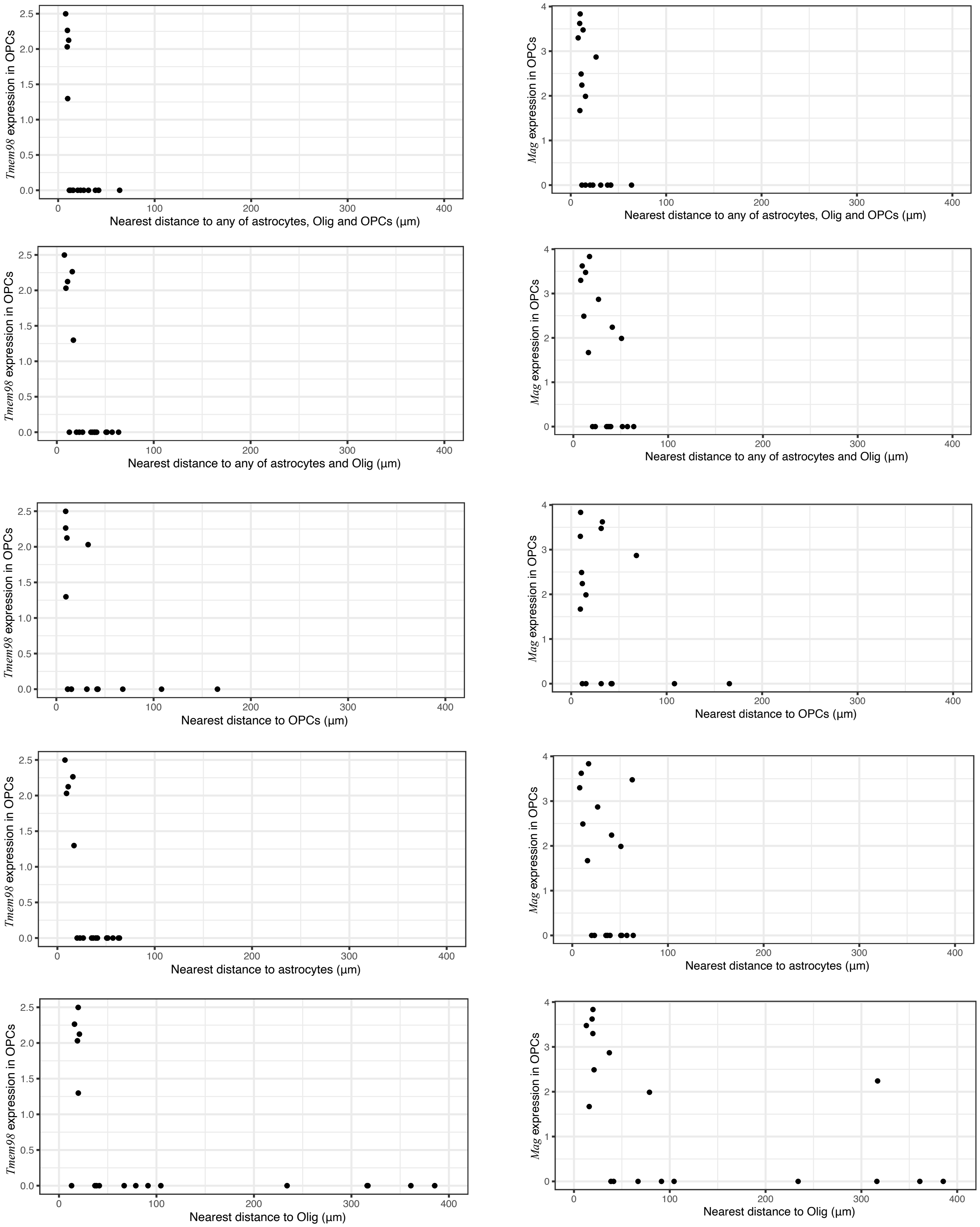

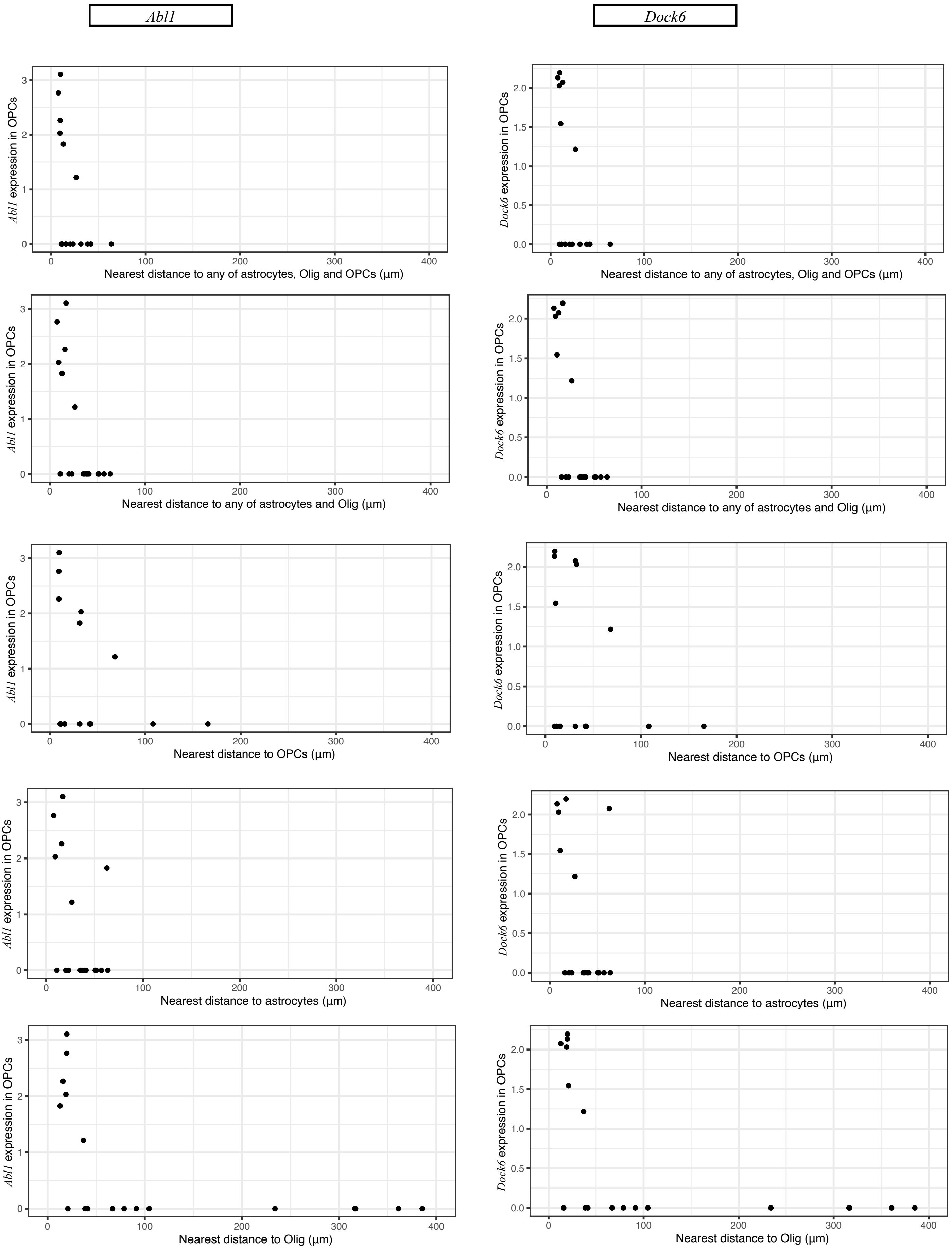

Abl1
Dock6
Abl1 expression in OPCs
Dock6 expression in OPCs
Nearest distance to any of astrocytes, Olig and OPCs (µm)
Nearest distance to any of astrocytes and Olig (µm)
Nearest distance to OPCs (µm)
Nearest distance to astrocytes (µm)
Nearest distance to Olig (µm)

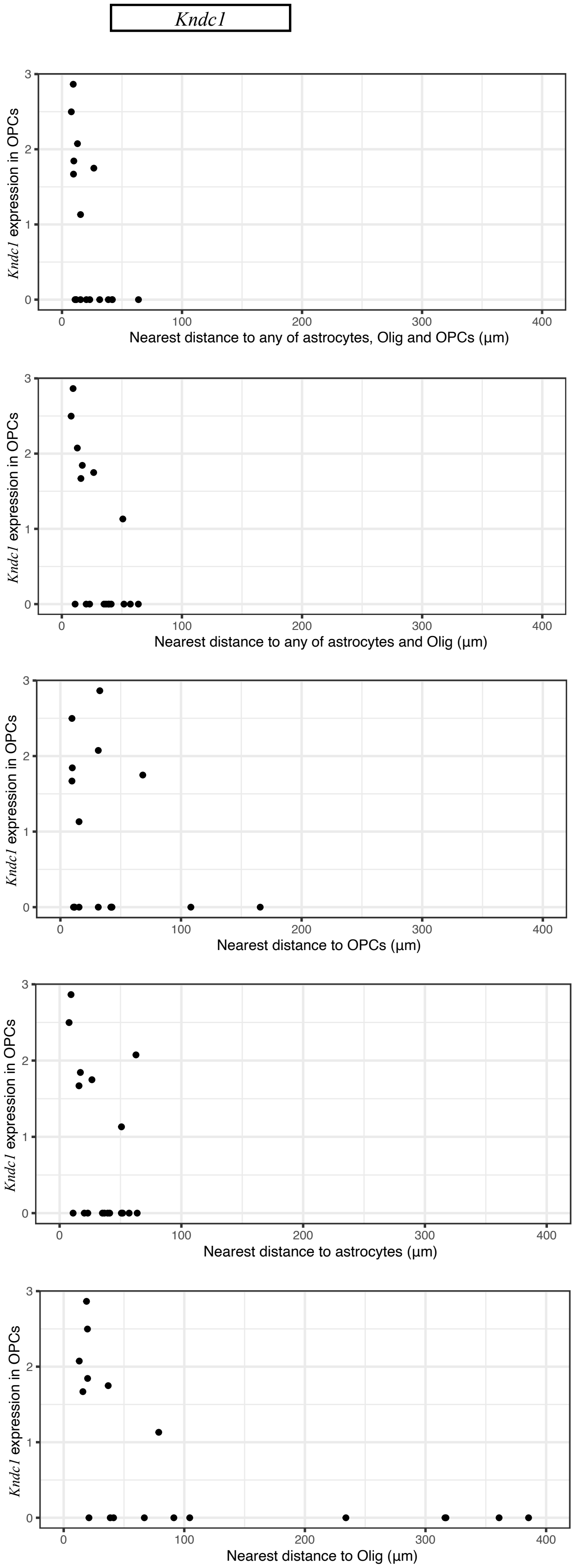

Figure S8. Relationships between the gene expression and the nearest distance to neighboring cell types. The indicated genes are the cluster 1 (*Tmem98*) and 2 (*Mag*, *Abl1*, *Dock6*, and *Kndc1*) genes in Oligodendrocyte Precursor Cells (OPCs). The x-axis indicates the nearest distance to one of the five patterns of neighboring cell types: (1) any of astrocytes, Oligodendrocytes (Olig) and OPCs; (2) any of astrocytes, Olig; (3) OPCs; (4) astrocytes; (5) Olig. The y-axis indicates the each gene expression in OPCs. The pixel scale in the original data was transformed into µm scale (each pixel is 106 nm).

Application to the seqFISH+ real dataset:

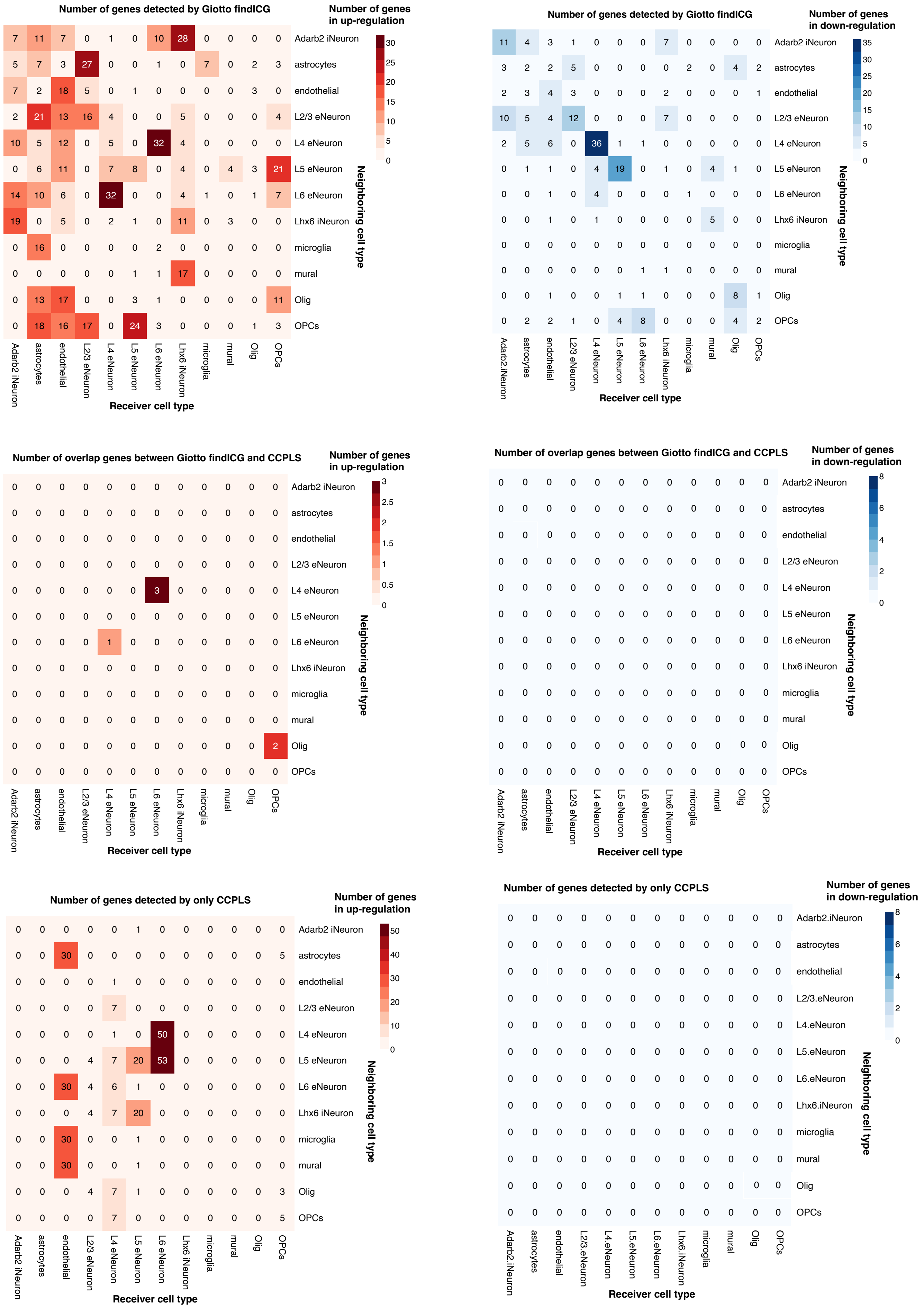

Figure S9. Comparison between Giotto findICG and CCPLS in the seqFISH+ real dataset. Note that no down-regulated genes were found for CCPLS (Fig. 3c and Supplementary Fig. S3).

Apllication to the seqFISH+ real dataset:

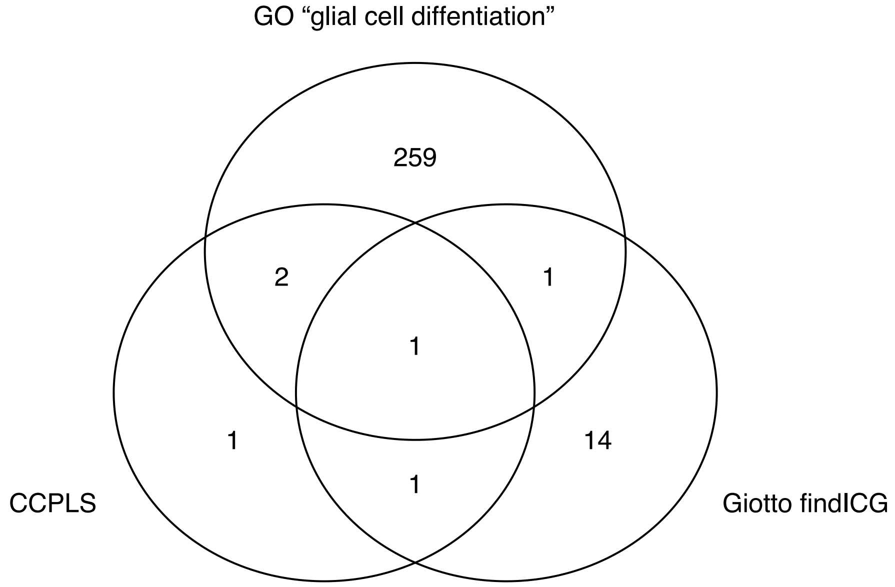

Figure S10. Number of overlaps between genes of GO ‘glial cell differentiation,’ genes detected by CCPLS, and genes detected by Giotto findICG. Note that we extracted genes in Oligodendrocytes Precursor Cells (OPCs) up-regulated by astrocytes, Oligodendrocytes (Olig), or OPCs as to CCPLS and Giotto findICG in this venn diagram.

Application to the Seq-Scope real dataset:

Color of heatmap: coefficient

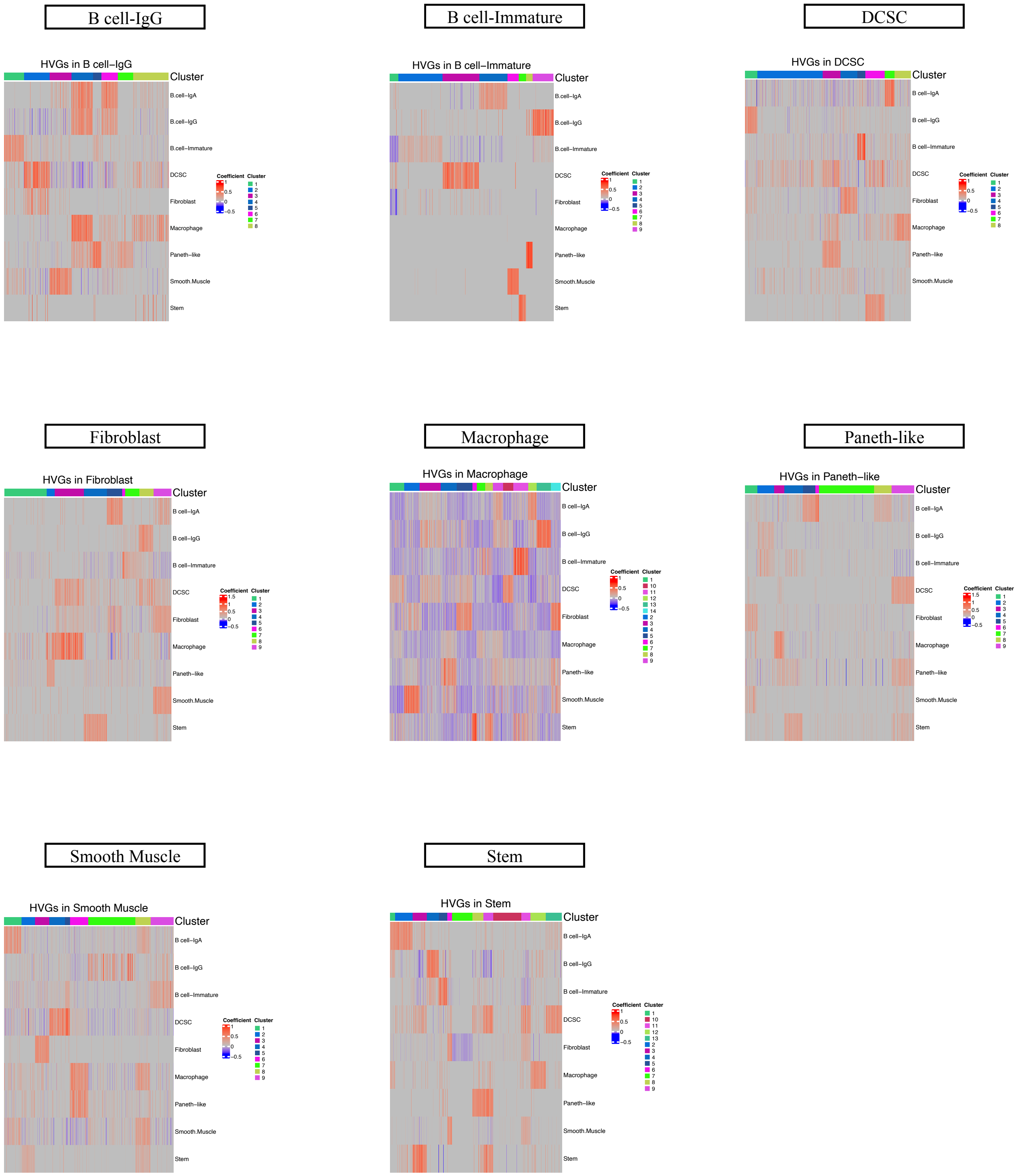

Figure S11. Heat map of all the cell types in the Seq-Scope real dataset. Rows and columns correspond to neighboring cell types and highly variable genes (HVGs), respectively. The color of the heat map indicates the coefficient. The heatmap of the immature B cell is the same as that in Figure 4c.

Application to the Seq-Scope real dataset:

Width of edge: averaged coefficients

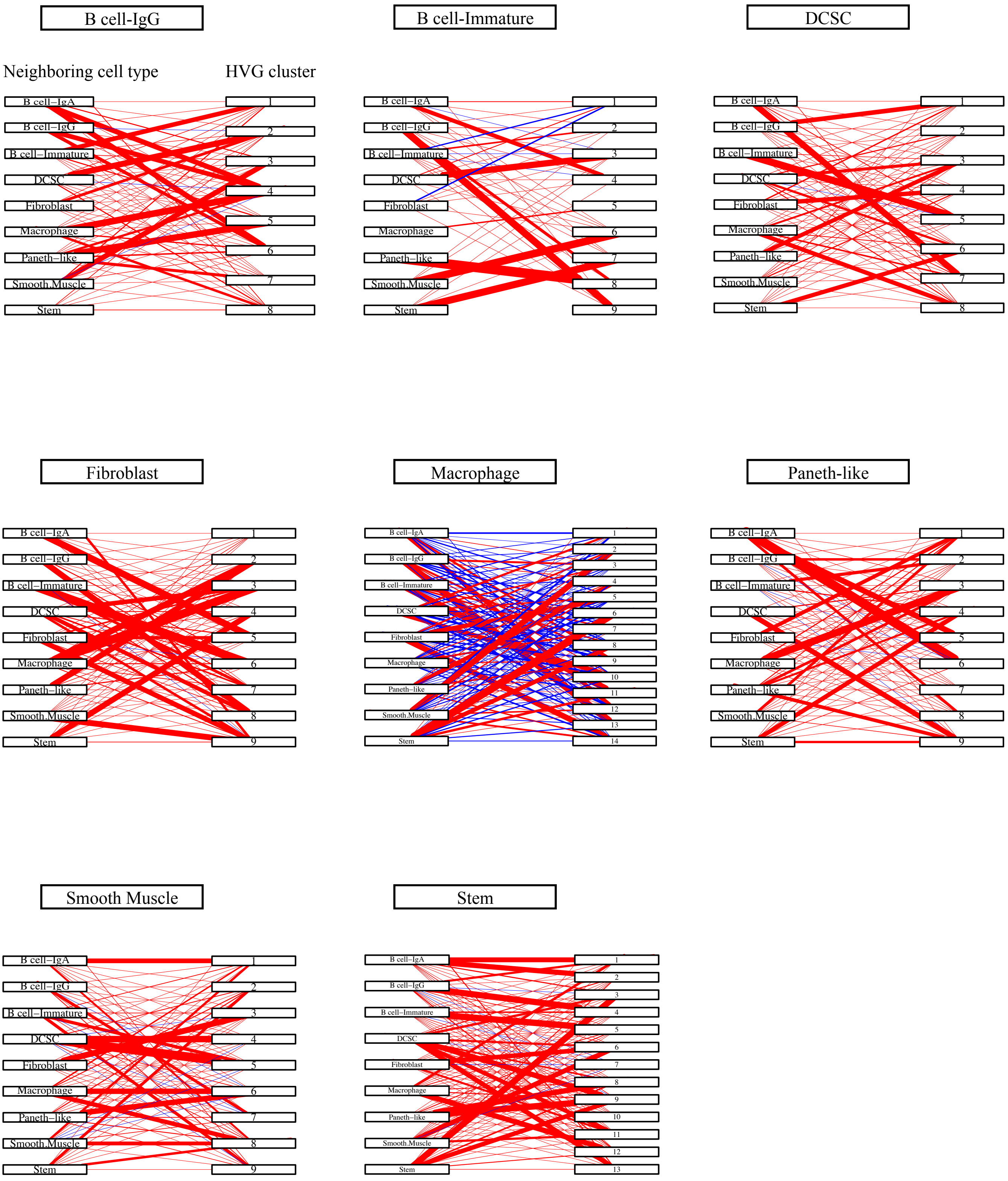

Figure S12. Bipartite graph of all the cell types in the Seq-Scope real dataset. The width of each edge indicates the averaged coefficients for each combination of highly variable gene (HVG) clusters and neighboring cell types. The bipartite graph of the immature B cell is the same as that in Figure 4d.

<u>Application to the Seq-Scope real dataset:</u>

Red: contributor cell type   Blue: non-contibutor cell typ   Row: neighboring cell type   Column: HVG cluster

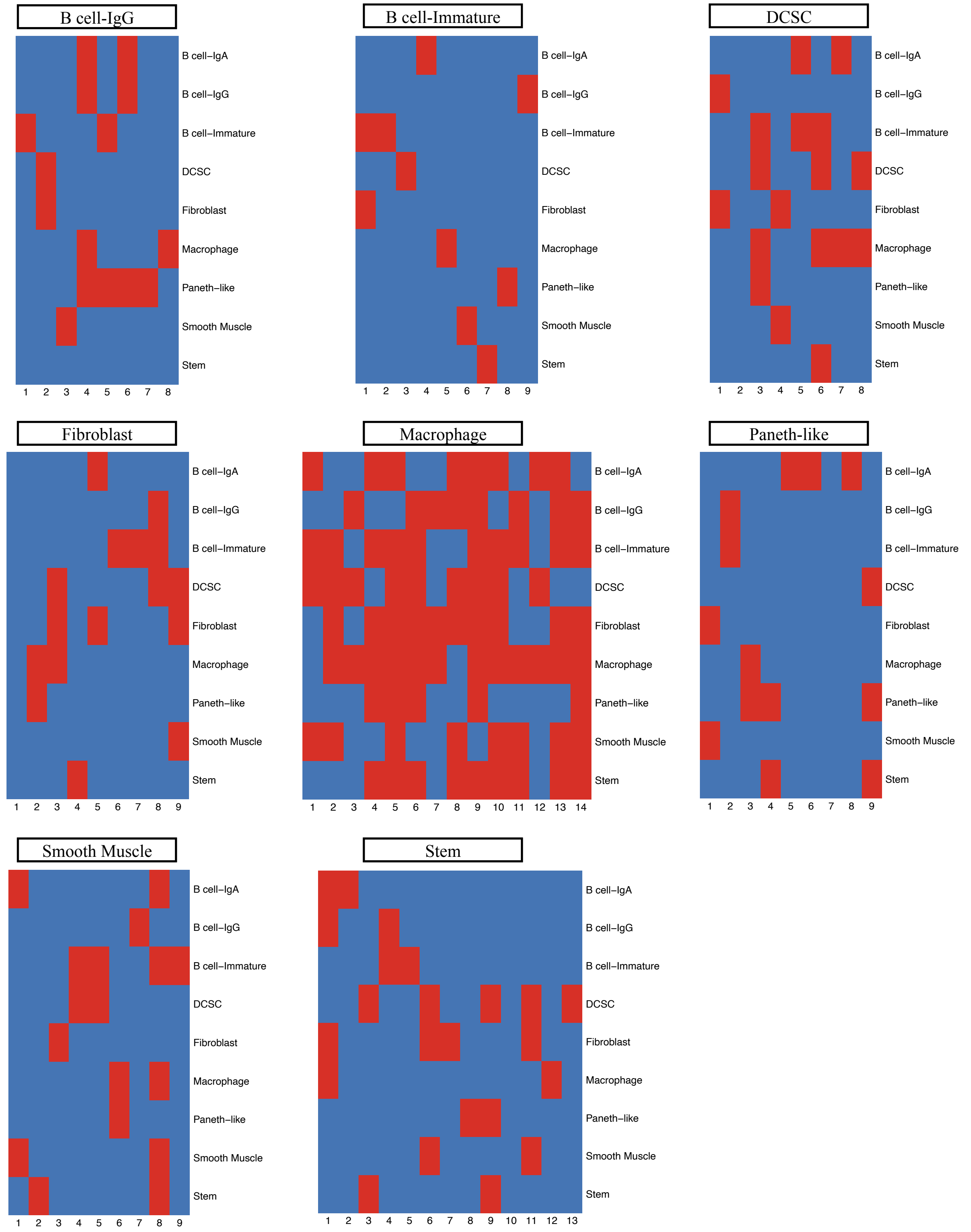

Figure S13. Classification of the contributor cell types in the Seq-Scope real dataset. The color of the heat map corresponds to the binary value indicating whether the neighbor cell type is a contributor cell type or not. Red and blue indicate the contributor and non-contributor cell types, respectively. Rows and columns correspond to cell types and highly variable gene (HVG) clusters, respectively.

Apllication to the Seq-Scope real dataset:

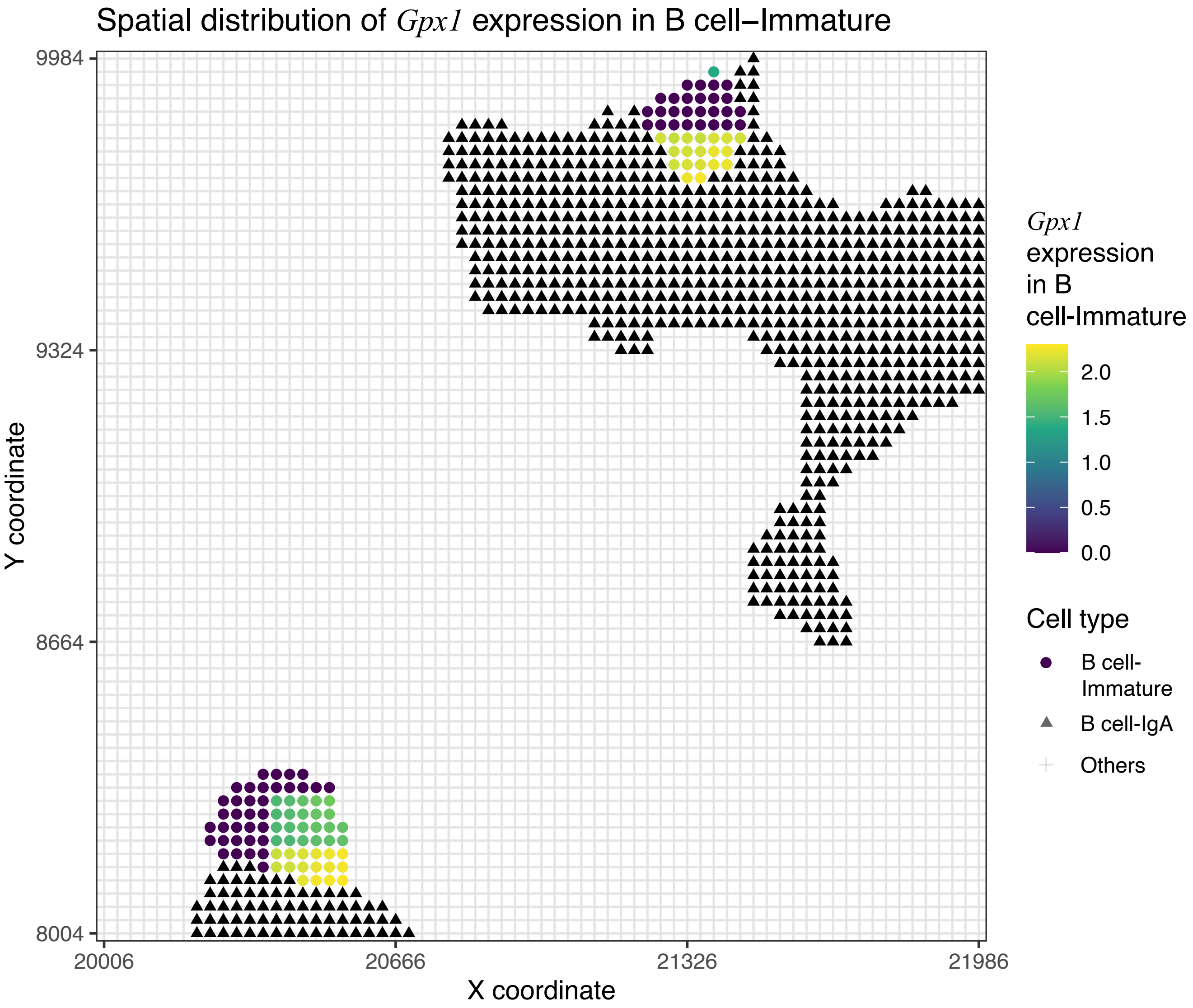

Figure S14. Spatial distribution of *Gpx1* expression in B cell-Immature. The shapes indicate cell types. The color in the circles indicates values of *Gpx1* expression.

Application to the Seq-Scope real dataset:

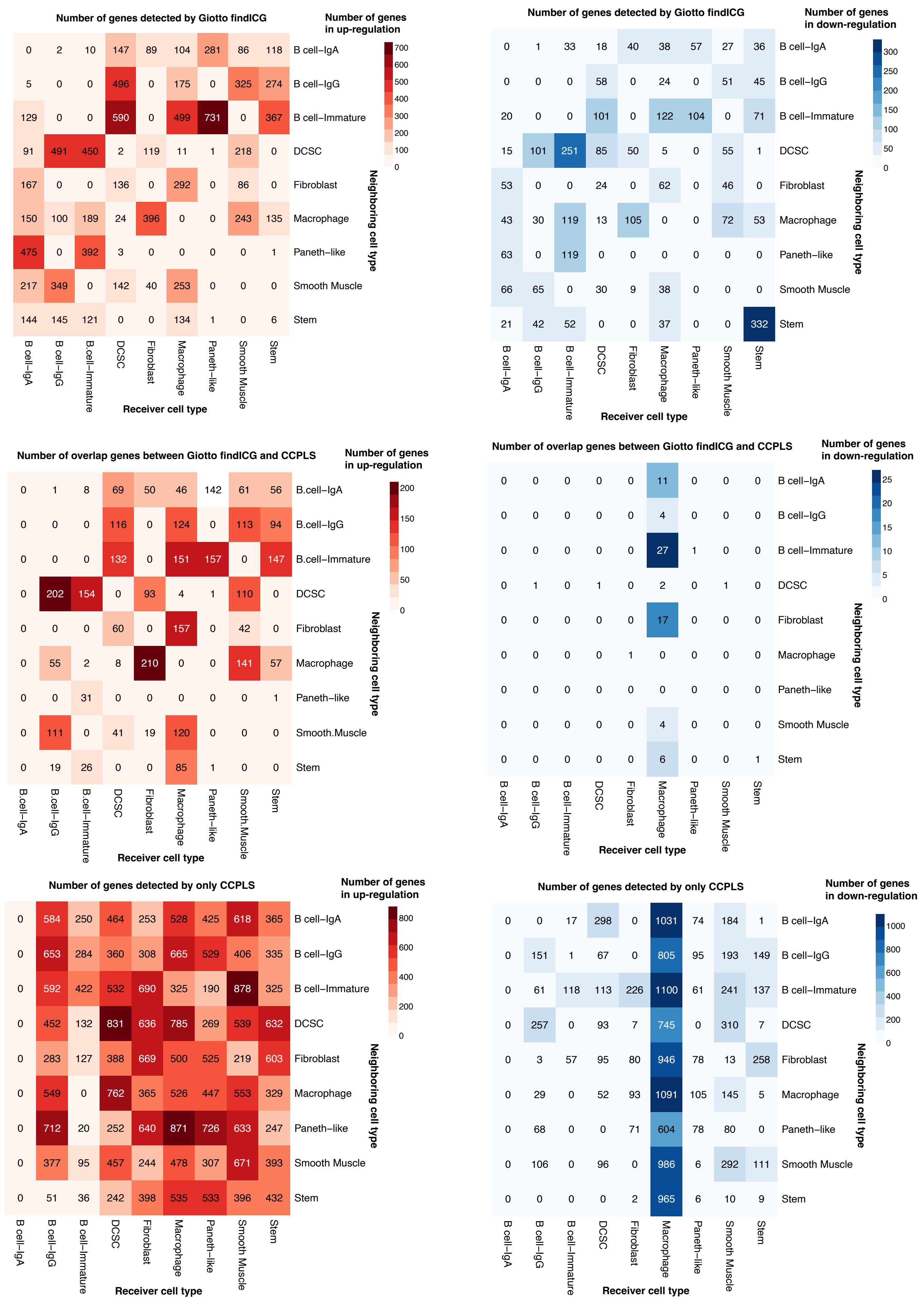

Figure S15. Comparison between Giotto findICG and CCPLS in the Seq-Scope read dataset.

Apllication to the Seq-Scope real dataset:

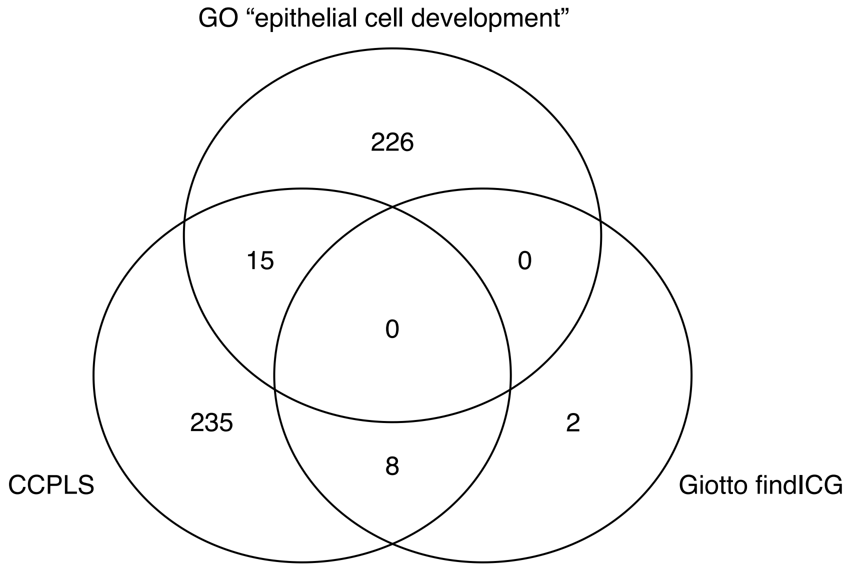

Figure S16. Number of overlaps between genes of GO ‘epithelial cell development,’ genes detected by CCPLS, and genes detected by Giotto findICG. Note that we extracted genes in immature B cell up-regulated by IgA B cell as to CCPLS and Giotto findICG in this venn diagram.

Application to the Seq-Scope real dataset:

Row: GO term Column: gene count Color of bar graph: adjusted p-value

B cell-Immature

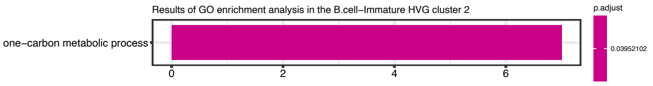

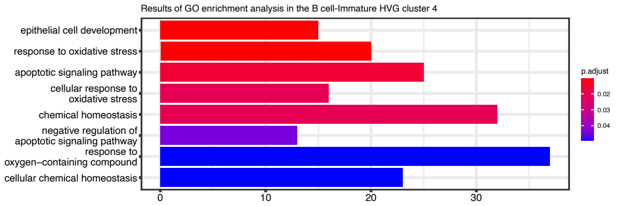

DCSC

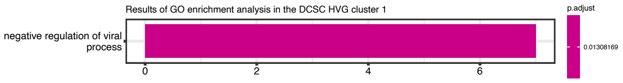

Fibroblast

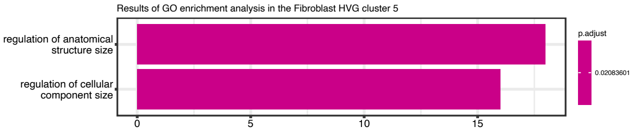

Macrophage

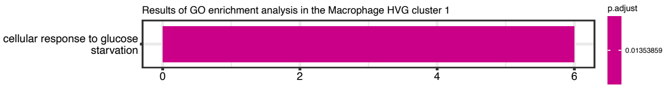

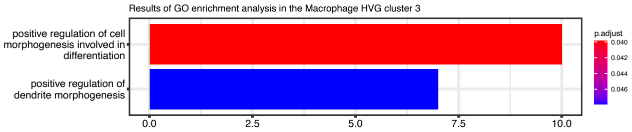

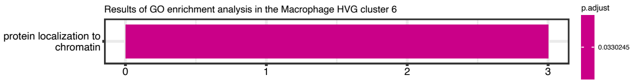

Stem

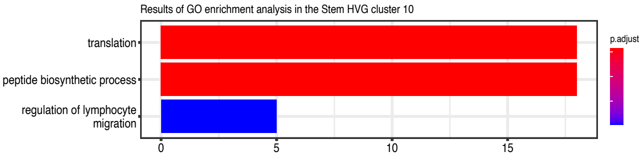

Figure S17. Gene Ontology (GO) enrichment of all the cell types in the Seq-Scope real dataset.
